# Implications of Using Host Response-Based Molecular Diagnostics on the Management of Bacterial and Viral Infections: A Review

**DOI:** 10.3389/fmed.2022.805107

**Published:** 2022-02-03

**Authors:** Johnny Atallah, Michael K. Mansour

**Affiliations:** ^1^Division of Infectious Diseases, Massachusetts General Hospital, Boston, MA, United States; ^2^Department of Medicine, Harvard Medical School, Boston, MA, United States

**Keywords:** infections, host-response, biomarkers, proteomics, transcriptomics, RT-PCR

## Abstract

Host-based diagnostics are a rapidly evolving field that may serve as an alternative to traditional pathogen-based diagnostics for infectious diseases. Understanding the exact mechanisms underlying a host-immune response and deriving specific host-response signatures, biomarkers and gene transcripts will potentially achieve improved diagnostics that will ultimately translate to better patient outcomes. Several studies have focused on novel techniques and assays focused on immunodiagnostics. In this review, we will highlight recent publications on the current use of host-based diagnostics alone or in combination with traditional microbiological assays and their potential future implications on the diagnosis and prognostic accuracy for the patient with infectious complications. Finally, we will address the cost-effectiveness implications from a healthcare and public health perspective.

## Introduction

The complexity of the human immune response in the setting of disease has made it difficult to assign the contribution from the underlying pathologic process in the background of the host immune response. The difficulty in such determination frequently leads to misdiagnoses, antibiotic misuse leading to antimicrobial resistance (AMR), increased healthcare expenses and direct adverse effects affecting the health of patients.

The clinical manifestations of pathogen-specific disease vary across a wide spectrum of symptoms including fever, myalgias, respiratory symptoms, weakness and altered mental status among many others. In fact, pathogen-based diagnostic testing has been the traditional and a convenient method for the identification of the causative pathogen linked with specific clinical manifestations, such as fever. This process is usually performed using traditional based culture systems, immunoassays, and molecular-based testing. Pathogen detection can usually be achieved by polymerase chain reaction (PCR) that can amplify the nucleic acid of pathogens directly from blood culture. However, a limitation to PCR is the necessity for a minimum pathogen burden in the bloodstream, which in turn results in several false-negative outcomes. Another limitation is the time constraint on laboratory staff performing repeat pathogen-based diagnostics in an attempt to improve sensitivity and detection.

The purpose of the immune system is to recognize and eliminate invading pathogens making a host response-based immunodiagnostic an attractive adjunct to pathogen-based diagnostics with the potential for improved diagnostics accuracy and efficiency. These techniques represent a step closer toward precision and personalized medicine capable of providing the best treatment matched for the specific patient in a timely manner (1). As such, the “omics” platforms have proliferated around host immunodiagnostics and several promising molecular host biomarkers show potential in the rapid diagnosis in critical diseases (2). Unlike pathogen-based testing, host immunodiagnostics present the capability of differentiating non-infectious immune triggers including sterile inflammatory processes, autoimmune diseases, or malignancy.

These techniques involve platform assays such as RT-PCR, RNA sequencing and others to test for specific host gene expression signatures and transcripts as well as metabolic and protein biomarkers directly related to susceptibility and response to infection. These technological advances have made it possible to integrate multiple biomarkers into single predictive models, and thus there is progress in the integration of genomics, transcriptomics, and proteomics with recent expansion into epigenomics, lipidomics, and metabolomics (3). While these approaches have the prospect of a more precise identification of an infectious trigger based on the host immune response, none to date have undergone clinical trial testing or achieved approval for clinical application.

Here, we review the current state of novel host response-based diagnostic testing on the identification of the causative processes underlying an activated immune response, on the influence on patient outcome, on reduction of healthcare cost, and on the possibility of redefining the standard of care for specific clinical presentations. This article will shed light on possible benefits of using host-based diagnostics from a public health perspective regarding pandemics and endemics, and finally, we examine techniques of integrating both, host-based and pathogen-based diagnostics for improving clinical outcomes.

## Methods

Publications on host immune response and role of immune based diagnostics were collected from the PubMed database. MeSH terms included host response, immune based diagnostics, transcriptomics, proteomics, infection, and sepsis were used to conduct this search. The articles were reviewed by the authors. Articles were limited to English language only and results were filtered by date of publication to include all articles published from 2015 through 2021.

## Results

### Host-Based Diagnostics for Identifying the Infectious Etiology

The initial management of suspected infection is pathogen identification, which subsequently dictates the treatment approach. In this section, we will review the use of host-based diagnostics in determining and identifying the infectious etiology. MeSH terms yielded 12 studies.

The host response to bacterial vs. viral test was examined. The study compared transcriptional analysis to a host immune biomarker, procalcitonin (PCT), which rises in the setting of bacterial but not viral infection (4). Results of the BioFire FilmArray system using RT-PCR to measure 45 transcript signatures were compared to standard PCT, yielded accurate discrimination between bacterial and viral infections superior to PCT performance. Six hundred twenty-three subjects with suspected respiratory infection or sepsis had blood testing for transcriptional profiling. The results provided 80.1% accuracy for bacterial infection and 86.8% accuracy for viral infection with a mean turnaround time of ~45 min compared to an accuracy of 68.7% for PCT alone (5). In addition to accurately detecting infectious processes, the BioFire FilmArray correctly identified ill patients without infection (no positive microbiology) with an 86% accuracy (6).

Several studies focused on using detection of host mRNA signatures to differentiate infectious from non-infectious processes in patients with acute infections and sepsis. The InSep^TM^ test (Inflammatix, Burlingame, CA, formerly known as HostDx^TM^ Sepsis) is a 29-host mRNA blood-based test that allows for rapid diagnosis of acute infections and sepsis using machine-learning algorithms. The patterns interpreted using InSep allows for differentiation of acute host response to bacterial vs. viral infections as well as prognosticating disease severity using whole blood. Following whole blood RNA extraction from patients with suspected sepsis in the emergency department, amplifying and quantitating the 29-mRNAs; these transcriptional signatures are then fed into machine learning algorithms to produce measurable scores. The 3 measurable scores (scale from 0 to 40) assess the likelihood of bacterial infection, the likelihood of viral infection, and the infection severity prediction score. However, one limitation is that some of the information presented was in some cases preliminary or hypothetical. An attractive feature of the InSep test is a rapid turnaround time of <30 min.

The 29 mRNAs that the InSep test consists of are classified into 3 separate, validated subpanels: a 7-mRNA “Bacterial-Viral Metascore,” an 11-mRNA “Stanford Mortality Score” and an 11-mRNA “Sepsis Metascore.” The 7-gene “Bacterial-Viral Metascore” subpanel consists of 4 genes (HK3, TNIP1, GPAA1, and CTSB) that have shown to be significantly higher in bacterial infections, and 3 genes (IFI27, JUP, and LAX1) shown to be higher in viral infections. The “Sepsis Metascore” subpanel on another hand, consists of a sepsis-specific transcripts including CEACAM1, C3AR1, GNA15, and HLA-DPB1 which have previously been linked to sepsis. Furthermore, neutrophil-related antimicrobial proteins genes such as DEFA4, CTSG, MPO, and BPI constitute the “Stanford Mortality Score” subpanel, along with additional genes related to energy metabolism and hypoxia (TRIB1, HIF1A, and NDUFV2).

Given the breadth of signatures included in the InSep platform, the potential exists to differentiate detection of bacterial or viral infection. The authors propose that application of RNA transcriptional analysis early in the presentation of a patient with a suspected infection reduces the ordering of multiple unnecessary diagnostics (7). The InSep assay showed a specificity of 98% and a sensitivity of 94% for detecting bacterial infections, and a specificity of 93% and a sensitivity of 96% for viral infections (8).

A similar platform using 29 host mRNA signatures analysis, a neural network classifier: Inflammatix-Bacterial-Viral-Non-infected-Version 1 (IMX-BWN-1) shows similar discriminatory results. The IMX-BVN-1 was used to assess patients with presumed infection and sepsis through the combination of mRNA host-response profiling combined with a machine learning algorithm. IMX-BWN-1 showed excellence diagnostic accuracy for bacterial and viral infection differentiation with a sensitivity of 97% and a specificity of 99%. The area under the curve (AUROC) for IMX-BWN-1 for identifying bacterial infections and viral infections was 0.87 and 0.86, respectively. The combination of mRNA expression analysis and machine learning proved superior to classic infection biomarkers such as PCT with an AUROC of 0.83 for bacterial infections and 0.27 for viral infections, and C-reactive protein (CRP) with an AUROC of 0.7 for bacterial infections and 0.38 for viral infections (9, 10).

In another pooled analysis of 1,057 samples from 20 cohorts, a set of 7 genes was derived for discriminating bacterial and viral infections. The 20 cohorts that were included either bacterial or viral infections, but not both. These cohorts represent a wide variety of clinical conditions, including a range of infection types (gram-positive, gram-negative, atypical bacteria, common respiratory viruses) as well as a range of severities (from mild infections to severe septic shock). This multicohort analysis aimed to use gene expression datasets for identifying a biomarker that can discriminate between viral and bacterial infections. Using this set alongside the 11-gene Sepsis MetaScore (Please see section “d” for more information) yielded a sensitivity of 94% and a specificity of 59.8% for identifying bacterial infections (11).

#### Infectious Etiology in the Pediatric Population

Infections are a leading cause for life-threatening events in the pediatric population. The WHO reports a global mortality rate of 5.9 million children under the age of 5 due to infections (12). Thus, host-response assays have emerged as promising diagnostics in this population.

In a prospective observational study febrile infants 60 days or younger were enrolled. The transcriptional assessment of 66 genes accurately identified infants with bacterial infections with a sensitivity of 87% and a specificity of 89%. Moreover, when 66 genes were reduced to 10 classifier genes, data continued to yield high diagnostic performance with a sensitivity of 94% and a specificity of 95% in distinguishing bacteremia in infants from those without infection as compared to confirmed bacterial blood cultures (13).

Furthermore, in a similar study, total blood RNA expression signature for distinguishing bacterial from viral infection in febrile children was compared with clinical and microbiological diagnostics. Subjects were classified into one of 3 groups: definite bacterial infection, definite viral infection and indeterminate state. These groups were stratified by culture or molecular detection of pathogens A two-transcript RNA signature (*FAM89A* and *IFI44L*) was identified from a larger 38-transcript screen. Then, the performance of a 2-transcript RNA signature expression was evaluated among the groups. The Family with Sequence Similarity 89 Member A (*FAM89A*) and the Interferon Induced Protein 44 Like (*IFI44L*) are both protein coding genes that have been linked to a rare mild immunodeficiency (immunodeficiency 38 with basal ganglia calcification). Upon implementation, this 2-transcript signature yielded favorable results for detection of definite bacterial with a sensitivity of 100%, and a specificity of 96.4% and definite viral with a sensitivity of 100%, and a specificity of 97.1%. IFI44L and FAM89A expression values were combined into a disease risk score. IFI4L was noted to be increased in antiviral responses mediated by interferons, while FAM89A was increased in bacterial infections and septic shock thus forming a reciprocal relationship of upregulation between both genes in viral and bacterial infections.

One interesting outcome was regarding the indeterminate groups where the 2-transcript signature detected 46.3% of those cases as having bacterial infection although 94.9% received antibiotic treatment by standard care (14, 15).

This 2-gene signature was further validated when applied to data from the RNA expression signatures used by the study described above. This validation study aimed to assess the accuracy of the 2-gene signature, previously tested in children with a mean age of 19 months, in infants aged 60 days or younger. The results were promising and showed a sensitivity of 88.8% and a specificity of 93.7% when compared to definite bacterial infections with positive cultures and confirmed viral infections. These data demonstrate the translatable potential of this 2-gene transcript signature into a simple bedside diagnostic test although a larger sample of subjects is needed for confirmation (16).

The application of technology amenable to bedside conditions show promise as a point of care RNA diagnostic. Use of reverse transcription-loop mediated isothermal amplification (RT-LAMP) technology demonstrated that the 2-gene RNA signature has the potential of being translated into a rapid and portable platform convenient for the use as a point-of-care test. A laboratory-on-a-chip platform that uses reverse transcription-loop mediated isothermal amplification (RT-LAMP) technology. RT-LAMP technology uses the mechanism of auto cycling strand displacement DNA synthesis using a polymerase with 2 pairs of primers used. Using 6 independent sequences at the start and 4 independent sequences toward the latter stages, RT-LAMP can recognize and amplify target sequences. This RT-LAMP uses numerous microsensors that can detect hydrogen ions released and thus detect changes in pH during NAAT under same experimental conditions of the previous studies (14–16). The results of translating this 2-gene signature to RT-LAMP were very similar to using microarray data used in the previous studies. Sensitivity and specificity were 100% for confirmed viral and bacterial infections. In addition to RT-LAMP platform being simple, the assay time required was <25 min which is considerably more rapid than microarray (17).

The application of RNA signatures to determine microbial composition and prognostic outcomes has been examined. In a retrospective study aiming to evaluate the use of microbial signatures of specific microbiota to prognosticate the severity of influenza virus infection, 36 pediatric (mean age of 3 years) subjects infected with influenza and presenting with symptoms for <2 days were recruited. RNA-gene sequencing, mNGS and computational analysis workflow were used to assess nasopharyngeal samples (NP) collected from these subjects. Results indicated that subjects having an increased bacterial diversity in their NP samples experienced milder disease. On the contrary, subjects with diminished abundance of *S. aureus* on one hand, and increased presence of Streptobacillus, Prevotella, Porphyromonas, Granulicatealla, Veillonella, Fusobacterium, and Haemophilus in their NP samples experienced severe respiratory or neurological influenza outcomes. These data demonstrate that use of RNA transcript as a reflection of microbiome diversity in the setting of influenza can potentially serve as an accurate prognostic indicator (18) (see Table 1).

**Table 1:** Use of host-response diagnostics for discrimination of bacterial vs. viral infections.

**References**	**Objective**	**Assay**	**Comparison**	**Genes**	**Sample size**	**Sensitivity**	**Specificity**	**PPV**	**NPV**	**Notes**
Tsalik et al. (5) de Jonge et al. (4)	Bacterial vs. viral discrimination	BioFire FilmArray system using RT-PCR	PCT	45 transcript signatures	623 adults with suspected respiratory infections	-	80.1% for bacterial 86.8% for viral 86% for no infection	-	-	Turnaround time of 45 min
Ducharme et al. (7) Safalika et al. (8)	Infectious vs. non-infectious discrimination	InSep Test using whole blood mRNA for host mRNA signatures	Traditional microbiology assays	29-host mRNA signatures	-	98% for bacterial 93% for viral	94% for bacterial 96% for viral	-	-	Turnaround time of 30 min
Mayhew (9) Bauer et al. (10)	Bacterial vs. viral discrimination	IMX-BWN-1 using whole blood mRNA for host mRNA signatures	Traditional microbiology assays + PCT + CRP	29-host mRNA signatures	1,069 adults with suspected infections	97%	99%	-	-	Performance superior to PCT and CRP
Sweeney et al. (11)	Bacterial vs. viral discrimination	Multicohort analysis using gene expression datasets to derive a biomarker		7-gene dataset	1,057 adults with suspected infections	94%	59.8%	-	-	-
Mahajan et al. (13)	Detection of Bacterial infections in febrile infants 60 days or younger	Transcriptional assessment of RNA biosignatures	Traditional microbiology assays	10-classifier genes	279 randomly selected febrile infants	94%	95%	-	-	-
Herberg et al. (15)	Bacterial vs. viral infection in febrile children	Microarray	Traditional microbiology assays and clinical assessment	2-gene transcript signature	455 children with fever	100% for bacterial 100% for viral	96.4% for bacterial 97.% for viral	-	-	The 2-transcript gene signature detected 46.3% of indeterminate subjects as having infection although 94.9% received antibiotics as per standard care.
Kafourou et al. (16)	Bacterial vs. viral infection in febrile infants <60 days old	Microarray	Traditional microbiology assays and clinical assessment	2-gene transcript signature	279 randomly selected febrile infants	88.8%	93.7%	-	-	Potential of being used as a simple bedside diagnostic test
Pennisi et al. (17)	Bacterial vs. viral infection in febrile children	RT-LAMP	Traditional microbiology assays and clinical assessment	2-gene transcript signature	455 children with fever	100%	100%	-	-	Turnaround time of 25 min significantly faster than microarray

Some limitations arise due to the special considerations of the pediatric population, which include difficulty of sample collection. In addition, some studies aimed to recruit equal numbers of children with confirmed bacterial and viral infections and then assess for diagnostic accuracy of the host-response assay. Thus, a limitation around possible bias in misrepresentation of infectious etiology and frequency in febrile children presenting to healthcare facilities.

### Host-Based Diagnostics for Identifying Respiratory Infections

#### Respiratory Infections

One of the most common causes of hospitalization and mortality in adults is lower respiratory tract infection (LRTI). Evaluation of whole blood gene expression profiling using RNA sequencing and qPCR for the discrimination bacterial from non-bacterial infection was performed. Using MeSH terms for host-based diagnostics for identifying bacterial vs. viral respiratory infections including tuberculosis yielded 13 studies.

Despite being a common cause of morbidity, mortality and hospitalization, LRTI-causing pathogens are infrequently identified due to limitations of traditional pathogen-based detection methods. In one study, an 11-host gene pathway set from nose and throat swabs, sputum, urine, and blood samples obtained from potential patients with symptoms of LRTI was used as an optimal marker. Quantitative PCR assay [e.g., Film Array Respiratory Panel, Idaho Technologies Inc. for nose and throat swabs (NTS) and sputum] was used for all the samples, and the difference in gene expression was tested by Wilcoxon Rank test. The Respiratory Panel offers a run time of about 45 min for rapid PCR detection of respiratory infections, and it integrates sample purification, amplification, detection, and analysis in one automated multiplex PCR system for detection of many pathogens within rapid time. RNA sequencing was also used and differences in gene expression between bacterial and non-bacterial infected subjects were assessed by a similar statistical approach. The results of this study showed promising outcomes with a sensitivity of 90% and a specificity of 83% for identifying bacterial LRTI as compared to confirmed microbiological testing (19).

Other studies have utilitzed metagenomic next-generation sequencing (mNGS) for DNA and RNA (see section “f” for more information) to define host signatures in response etiologic pathogens resulting in LRTI. In a prospective observational study comparing mNGS from patients with and without LRTI to traditional assays, this novel host-based platform detected more viruses and fungi and at a more rapid rate with an approximate 2-day turnaround time. It showed a positive predictive value (PPV) of 78.5%, sensitivity of 66.7% and specificity of 75.4%. Such results will provide insight regarding the impact of the host transcriptome data in the accurate diagnosis of LRTI (20).

In addition to PCR and transcriptional analysis, circulating host biomarker have also been explored as diagnostic and prognostic indicators of infection. One such molecular is proadrenomedulin, a receptor expressed on myeloid cells showing encouraging results for predicting complicated community acquired pneumonia (CAP) in the pediatric population. Proadrenomedullin is a member of the calcitonin peptide family that has been shown to be expressed proportionately during severe infections and is widely expressed by many tissues and organs. It increases microvasculature flow to maintain adequate vascular supply to vital organs during sepsis (21). A proadrenomedullin level above 0.16 nmol/L generated using TRACE (time-resolved amplified cryptase emission) showed a sensitivity of 100% and a specificity of 70% for bacteraemia in children (0–18 years of age) presenting with community acquired pneumonia (22, 30).

The evaluation of proadrenomedullin in the assessment of adult patients with CAP shows similar results when compared to pneumonia severity index (PSI) and CURB65 scores, as a prognostic indicator. Eighty-one patients with suspected CAP were enrolled and followed up to a 28-day duration. Results showed an increased prognostic accuracy for CAP when CURB65 scores were used in combination with proadrenomedullin levels. In fact, for the highest risk patients with upper score classes of PSI and CURB65, proadrenomedullin levels provided additional risk stratification. This result provided valuable accuracy and guidance to the patients' need for intubation, non-invasive ventilation and ICU admission. Using specific proadrenomedullin levels for predicting outcomes yielded a sensitivity of 77.8% and a specificity of 76.5% for death when the value is 1.6 nmol/L, a sensitivity of 83.3% and a specificity of 88.7% for endotracheal intubation when the value is 2.4 nmol/L, and a sensitivity of 87.5% and a specificity of 77% for non-invasive mechanical ventilation at a value of 1.5 nmol/L (31).

#### Coronavirus Disease 2019

SARS-CoV-2 is the causative respiratory viral pathogen responsible for the COVID-19 (32). Given the need for rapid diagnostics, multiple studies explored the use of host-based diagnostics for the detection of COVID-19. In one study, the aim was to derive a transcriptional signature to detect multiple viral infection among including COVID-19. Whole-blood RNA sequencing on samples from subjects was performed with confirmed bacterial, viral or no infection cases. Signature host genes were derived and validated using RT-qPCR. Three-signature genes (*IGF1R, NAGK*, and *HERC6*) were derived from the subjects enrolled by differential gene expression analyses using forward selection-partial least squares. The *IFG1R* represents an insulin signaling tyrosine kinase protein that has shown to act as an entry receptor for respiratory syncytial virus (RSV) as well as macrophage and phagocytosis activation. *NAGK* is an enzyme responsible for amino acid metabolism, and *HERC6* has been reported to have antiviral activity when induced by interferon. These gene transcripts distinguished bacterial from viral infections with a 97.3% sensitivity and 100% specificity with superior performance to CRP and leukocyte count. A second validation analysis was done, and the 3 gene signature distinguished between bacterial and COVID-19 positive subjects with a sensitivity of 88.6% and a specificity of 94.1% also outperforming CRP levels and leukocyte count (23).

In one recent study of COVID-19 infected subjects, RNA-sequencing was used to assess the host response in nasopharyngeal and whole blood samples. This technique allowed the derivation of a 19-gene host-response classifier that can differentiate COVID-19 infection from other infections with an accuracy of 86.5%, sensitivity of 80% and specificity of 90% using NP samples. The dysregulated immune response with COVID-19 showed a distinct pattern of activation and inhibition of immune pathways as compared to other infections such as influenza, seasonal coronaviruses, and bacterial sepsis. Moreover, the magnitude of the host-response was found to be directly proportional with clinical severity of the disease. Remarkably, an increased expression of genes involved in interferon responses and decreased expression of IL-6 and IL-18 signaling was noted. Other genes such as ACE2 and TMPRSS2 have shown an association with the need of oxygen therapy during COVID-19 as well as predicting disease severity. However, these genes did not necessarily prove to be upregulated in COVID-19, whether from whole blood or nasopharyngeal swab. The results show that the expression of both genes can serve a prognostic rather than diagnostic role (29, 33).

Such a study points out to the potential of using classifiers of host-response for diagnosis of COVID-19 in the pre-symptomatic or asymptomatic stage during which 38% of pathogen-based PCR will turn out negative (33).

#### Influenza and Respiratory Syncytial Virus

Influenza virus, known as “the flu” is one of the most common seasonal respiratory infections worldwide (34). The average pre-symptomatic incubation period of influenza is 2 days. Oseltamivir, a neuraminidase inhibitor, is a therapeutic intervention used in the pre-symptomatic phase shows reduction in the progression of disease, decrease symptoms, infectivity, and accelerated resolution of disease. Early identification of influenza-infected individuals would permit more effective use of antiviral interventions. The use of host-based immune response for early detection of influenza was examined including the implications on management and therapy. Subjects were intranasally inoculated with influenza A and host gene expression was then assessed in peripheral blood samples every 8 h for 7 days using the GeneChip Human Genome U133A Array (Affymetrix, Santa Clara, CA), which is a single array representing 14,500 genes. This process led to the derivation of a gene signature expression for influenza virus composed of 50 genes. The host inflammatory response represented by the gene signature derived was then monitored after the early therapeutic use of oseltamivir in inoculated subjects. It was noted that the markers of host response were significantly reduced upon early treatment with oseltamivir demonstrating a correlation between disease activity, symptoms over time and overall expression of gene-signature levels. The level of host-gene expression was in agreement with the trajectory of symptom progression, thus showing the significance of the impact of time on host-response diagnostics. Although the application of such a technology is complex, the use of a potential rapid and accessible platform (i.e., PCR-based assays) as described in this article could help overcome this limitation. This study is important for providing insight on the correlation of disease severity and gene signatures as well as demonstrating the temporal dynamics of genomic signatures and their response to early treatment (24).

The number of gene biomarkers required has also been examined. A single gene biomarker, IFI27, was used for discriminating between influenza and bacterial infections was identified using integrated genomic analysis. *In vitro* experiments have shown that IFI27 was expressed by antigen presenting cells responding to influenza virus. *In vivo* studies confirmed expression of IFI27 in influenza patients. In fact, in this prospective study enrolling patients with suspected respiratory illness, IFI27 showed high diagnostic accuracy of 88% and a specificity of 90% for distinguishing between influenza and bacterial infections equivalent to accuracy obtained by using multi-gene biomarkers (25).

Although IFI27 has demonstrated the potential of differentiating influenza virus from bacterial infections, other studies using the same gene marker in the context of other viral respiratory infections show similar results. In one study of preterm RSV-infected infants, IFI27 was highly expressed, and its expression correlated with the severity of the disease (35).

Moreover, in another multi-cohort observational study, IFI27 was shown to be expressed in COVID-19 infected patients, and its level of expression was associated with the presence of a high viral load (36). These results are promising although further validation is required to achieve high specificity of this gene marker to a particular disease.

Since IFI27 has been found to be upregulated in influenza, RSV and COVID-19, an effort to identify a single-gene biomarker with a high diagnostic accuracy and specificity to influenza virus in one study was attempted. XGBoost integrated bioinformatics analysis was used to identify 14 genes specifically related to influenza infection using data from obtained from the gene expression Omnibus database. One gene, oligoadenylate synthetases-like (OASL), was further identified from the 14 gene set and was shown to differentiate between influenza and non-influenza viral and bacterial respiratory infections sharing comparable clinical features outperforming IFI27 with an AUC of 0.85 vs. 0.76, respectively. OASL is known to possess antiviral mediated roles and has been recently shown to have a role in antiviral innate immunity, and it has been previously studied in the context of differentiating viral from bacterial infections. However, OASL's expression value measured by qRT-PCR can be sufficient to differentiate influenza from other non-influenza viral infections. Thus, this study presented significant results to identify OASL as a single biomarker for accurate and specific influenza virus identification (37).

Host-response profiling is not limited to diagnostic potential but also for predicting disease severity. In a study of RSV, the association between nasopharyngeal microbiota and host response profiles predicted the disease severity in RSV-infected children. Nasopharyngeal microbiota was characterized from children with mild and severe RSV using RNA sequencing. In turn, whole blood transcriptome profiles were analyzed to find the potential relationship between the microbiota, RSV host response and consequently, disease severity. RNA from whole blood was hybridized onto Illumina HT12-V4 bead chips.

The data revealed different nasopharyngeal microbiota clusters correlated with interferon related genes from the host response to RSV infections. A significant result overexpression of interferon genes related to neutrophil and macrophage activation in RSV infected children with *H. influenza* and Streptococcus dominant microbiota. This provides a demonstration of the possible interaction between the nasopharyngeal microbiota and the host response in RSV infected children ultimately in determining disease severity (35, 38).

A multi-cohort analysis approach for exploring host transcriptome biomarkers to derive a transcript-gene signature was undertaken as a better RSV diagnostic. Meta-analysis of 7 transcriptome microarray studies consisting of 922 whole blood samples from RSV, healthy, coronaviruses, rhinoviruses infected adults and children identified over 1,500 expressed genes from RSV-infected patients. Furthermore, selectively studying various pathways significantly affected by RSV yielded a 17 transcript host gene signature that is specific for RSV and can differentiate it from other respiratory infections. The results showed a sensitivity of 81.3% and a specificity of 93% for distinguishing RSV from other viral infections using this 17-transcript host signature (26).

In a similar manner, one study used whole blood mRNA signatures to assess the severity and pathogenicity of influenza virus. Certain signatures related to interferon antiviral pathways proved to be common in influenza cases not requiring intubation. As for those requiring mechanical ventilation support, inflammatory, activated neutrophil pattern was seen as early as possible in the course of the disease. Thus, using host-based profiling can potentially project the clinical course of influenza and provide insight on therapeutic tools for severe cases (38, 39).

### Host-Based Diagnostics for Identifying *Mycobacterium tuberculosis*

*Mycobacterium tuberculosis* (*M. tuberculosis*) is a potentially life-threatening infectious disease with typical pulmonary primary infection. The use of host-immune based diagnostics to support the identification of *M. tuberculosis*, disease severity and treatment response was assessed. The focus of these novel diagnostic models was on the ability of improved sensitivity for the detection of smaller disease signatures with higher discriminatory power (40).

The World Health Organization (WHO) identified the need for non-sputum-based diagnostic tests for better diagnosis of *M. tuberculosis* and for differentiating active from latent disease states. The need for new non-sputum diagnostics for active *M. tuberculosis* is realized by the difficulty and poor sensitivity of traditional growth-based microbiology approaches. In an integrated multicohort analysis of existing gene expression microarray from peripheral blood of patients with active *M. tuberculosis* composed of 2,572 patient samples, deriving a diagnostic gene set was attempted. Patients with latent *M. tuberculosis* and other diseases (i.e., sarcoidosis, autoimmune infections, lung cancer) were compared to those with active *M. tuberculosis* using the available multicohort analysis framework. Following analysis, a three gene set out of 266 demonstrated significantly higher diagnostic accuracy for active vs. latent *M. tuberculosis* from whole blood. These 3 genes were *GBP5, DUSP3*, and *KLF2*. *GBP5* is a protein coding gene known to activate inflammasome assembly and reported to have a role in innate immunity and inflammation. Similarly, *DUSP3*, a protein phosphatase, and *KLF2* play a role in modulating innate immunity. This dataset distinguished active *M. tuberculosis* from healthy subjects with a sensitivity of 93% and a specificity of 97%. Such a gene set could potentially offer a framework for better diagnosis and treatment response to active *M. tuberculosis* (27).

In a similar study, published gene signatures for active *M. tuberculosis* diagnosis were identified using unbiased screens. Sixteen gene signatures were found. Twenty-four datasets containing 3,083 transcriptome profiles from whole and peripheral blood of healthy, active *M. tuberculosis*, latent *M. tuberculosis* and other diseases subjects were screened. A similar conclusion was made with the 3 signature genes (*GBP5, DUSP3*, and *KLF2*) described above demonstrating significant discrimination in identifying subjects with active *M. tuberculosis* and in predicting those with high risk of progression from latent to active *M. tuberculosis* with a sensitivity of 90%. These results demonstrated superiority over traditional sputum tests with a sensitivity of 53.3% (28).

This three-gene *M. tuberculosis* score was further tested in a cohort study for performance, not only as a diagnostic, but as an indicator for *M. tuberculosis* treatment response and on post-treatment residual inflammation. The three-gene *M. tuberculosis* score detected patients with active *M. tuberculosis* with a negative predictive value (NPV) of 99.3% at a prevalence of 4%. Additionally, with a sensitivity of 86% and a specificity of 84%, the three-gene mRNA expression score measured by qPCR or RNA sequencing showed accurate diagnosis of progression of latent to active Tb with an 86% sensitivity and 84% specificity, 6 months earlier than traditional sputum conversion which has a lower sensitivity of 45–61% (41) (see Table 2).

**Table 2:** Results of using host-response diagnostics for identifying respiratory infections.

**References**	**Objective**	**Assay**	**Comparison**	**Genes**	**Sample size**	**Sensitivity**	**Specificity**	**PPV**	**NPV**	**Notes**
Bhattacharya et al. (19)	Identifying bacterial LRTI	PCR assays and RNA sequencing	Standard of care	11 gene pathways	94 adults with suspected LRTI	90%	83%	-	-	Turnaround time of 45 min
Chen et al. (20)	Diagnosing LRTI	mNGS	Traditional microbiological assays		162 adults with and without LRTI	66.7%	75.4%	78.5%	-	-
Alcoba et al. (21) Saleh et al. (22)	Diagnosing bacteremia in children (0–18 years old) presenting with community acquired pneumonia	TRACE	Traditional microbiological assays	Proadrenomedullin levels	88 children	100%	70%	-	-	-
Li et al. (23)	Diagnosing COVID-19	RT-qPCR	CRP and leukocyte count	3-gene transcript signature	228 adults	88.6%	94.1%	-	-	
McClain et al. (24)	Early detection and treatment of influenza (in the pre-symptomatic phase)	GeneChip Human Genome U133A Array (microarray)	Standard methods	50-gene signature	21 healthy adults inoculated with influenza	-	-	-		Demonstrating temporal dynamics between gene signatures and early treatment
Tang et al. (25)	Influenza vs. bacterial infections	Integrated genomic analysis	Standard methods	1-gene (IFI27)	1,071 individuals	88%	90%	-	-	Diagnostic accuracy of this 1 gene signature equivalent to using multi-gene biomarkers
Barral-Arca et al. (26)	Diagnosing RSV infection	Meta-analysis of 7-transcriptome microarrays from whole blood samples		17-transcript host genes	922 samples	81.3%	93%	-	-	-
Sweeney et al. (27)	Non-sputum host-based diagnostics for active Tb	Integrated multicohort analysis of existing gene expression microarray from peripheral blood	Traditional growth-based microbiology diagnostics	3-gene signature	2,572 patients	93%	97%	-	-	-
Warsinske et al. (28)	Using the 3-gene signature in Rossi et al. (29) for studying treatment response and progression of latent to active Tb	qPCR and RNA sequencing	Traditional sputum conversion	3-gene signature	363 subjects	86%	84%	-	99.3%	This assay showed accurate diagnosis of active to latent Tb progression 6 months earlier than traditional sputum conversion

Moreover, soluble protein biomarkers such as interferon-inducible protein 10 (IP-10) have shown high sensitivity (98%) and specificity (87%) for Tb infection with superior sensitivity compared to interferon gamma-based IGRA test (42). In fact, in a recent study of *M. tuberculosis* infection, the aim was to identify host biomarkers for discrimination between latent and active *M. tuberculosis*. Using PCR assays on serum and saliva samples from active *M. tuberculosis* patients and their contacts, numerous chemokines, cytokines, and growth factors were assessed. Results were favorable for differentiating latent and active *M. tuberculosis* using interferon-inducible protein 10 IP-10 and B-Cell attracting chemokine (BCA-1) in serum with an AUC of 0.83, specificity of 88% and sensitivity of 72%. Moreover, testing for IP-10 in saliva showed an AUC of 0.68, sensitivity of 52% and specificity of 68%. This provides additional insight on the role of host-response diagnostics on differentiating latent vs. active *M. tuberculosis* infections (43, 44).

### Host-Based Immunodiagnostics in Sepsis

Host immune-based diagnostics have also been studied in sepsis, a potentially life-threatening process in the setting of serious infections. Using MeSH terms for sepsis, host-response, and infections we have found 5 citations of studies.

The InSep test (previously mentioned in section “a”) provides better insight to guide decision making. The host data, reflecting activation of immunity, can offer more real-time guidance for antimicrobial stewardship programs in management of appropriate antibiotic usage reduction of antimicrobial resistance and drug side effect. In addition, the rapid turnaround time allows for efficient diagnosis of sepsis along with determination of prognosis and disease severity (7, 8).

Multiple clustering analysis from host transcriptomics in a retrospective study of patients with bacterial sepsis revealed three robust clusters. These subtypes were derived from a unified clustering analysis across 14 discovery datasets. The three robust clusters were termed “Inflammopathic,” “Coagulopathic,” and “Adaptive.” Such clusters represent the heterogeneity of sepsis, and each subtype is associated with different mortality rates and different clinical coagulopathy rates. The “Inflammopathic” cluster was associated with higher mortality and an innate immune activation; the “Coagulopathic” cluster was associated with higher mortality, older patients and evidence of coagulopathy, and the “Adaptive” cluster showed an association with lower mortality and adaptive immune activation. These results represent a broad definition of the host-response to sepsis (45).

In a similar manner, studies using single-cell RNA sequencing of peripheral blood from subjects with sepsis defined 16 immune cell states. Using monocytes and dendritic cells, the outcome attained was identification of a sepsis specific CD14+ monocyte state. This monocyte state has specific surface markers and ultimately demonstrates that use of single-cell RNA sequencing can lead to the identification of unique disease associated cytologic signatures in bacterial sepsis (46).

Sepsis is a process that is not just limited to the adult population; in fact, neonates are at increased risk for developing sepsis. The complexity and ambiguity of the neonatal immune response has made it difficult to diagnose infections. There is no single biomarker that has yet proven to perform with sufficient accuracy for ruling out pediatric sepsis. Using host whole blood expression for 11 gene (Sepsis MetaScore, company, city, state), pediatric patients with sepsis were evaluated. The Sepsis MetaScore showed higher accuracy in diagnosing sepsis among 3 cohorts of neonates from several different countries as compared to standard neonatal lab tests. The sensitivity and specificity were 95 and 60%, respectively, as compared to standard microbiological testing with a sensitivity of 70% for a leukocyte count>15,000 and <3,000, and a sensitivity of 90% for CRP>10 mg/L. As for adults, implementing such improved diagnostics would lead to less AMR as well as decreased neonatal mortality rates (47).

### Effect of Host-Based Diagnostics on Healthcare Cost and Public Health Measures

The impact of host-based diagnostics has also been studied economic and public health outcomes in four studies.

Host-based immunodiagnostics were used to examine high risk close contact exposures. Participants who were in proximity of patients diagnosed with a respiratory viral infection were recruited, and a blood based 36 gene RT-PCR assay as a transcriptomic biomarker was used in an attempt for early identification of viral infection. The results were promising and have shown that such an assay can serve as an accurate prediction for viral infection at both the time of maximum symptom severity as well as up to 3 days before symptoms arise when compared to definite viral infection confirmed by PCR. This transcriptomic assay predicted viral infection at the peak symptom severity with an AUROC of 0.94, at 1, 2, and 3 days before symptoms arise with an AUROC of 0.87, 0.85, and 0.74, respectively. This study was the first real-world study to show that a host gene expression-based assay can accurately predict a respiratory viral infection before typical symptoms are present (48).

From an economic point of view, HostDx™ Sepsis (Inflammatix, Inc., city, state), a multi-RNA host response expression platform, was compared to the standard of care including procalcitonin. Results showed substantial reduction of average cost estimated to be around a $1974 USD per patient. Excluding the cost of the test itself, this overall healthcare cost reduction was attributed to a shorter stay at the hospital, decrease mortality rates at 30 days and less antibiotics being prescribed (7, 49). Additionally, a decline in the number of blood cultures drawn can be achieved, as well as mechanical ventilation and ICU stay days.

Moreover, platforms utilizing two-gene transcript RNA signature translated to RT-LAMP can prove to be cost-effective (due to absence of fluorescent label) with an average assay cost of, $1.33 USD per chip (17).

### Integrating Host-Based Diagnostics With Pathogen-Based Testing for Improved Clinical Outcomes

Despite the recent rise attention on host-based diagnostics, pathogen-based diagnostics continue to be the gold standard and the most frequently used assays for infectious disease detection. Therefore, being able to integrate host-based with pathogen-based diagnostics for increased sensitivity and better outcomes is an area of active investigation.

In a prospective cohort study of critically ill patients with acute respiratory failure, a combination of three elements: pathogen, host gene expression signatures and the airway microbiome using a developed sequencing-based approach was studied. The hypothesis of the study states that the combination of host response testing with simultaneous detection of possible respiratory pathogens and measurement of lung microbiome diversity could serve as a more precise and accurate platform for infection. In the host-response, upregulation of pathways related to 414 expressed genes was shown in the LRTI patients. These sets of transcriptional signatures differentiated LRTI subjects from the non-LRTI group which showed another set of upregulated pathways. On the other hand, the LRTI prediction using pathogen diagnostics was based on a logistic regression model. A logistic regression model microbial score was derived to classify subjects as having lower respiratory tract infection or not. The third element was lung microbiome diversity, and the rationale based on several studies is that a reduction in the diversity of the airway microbiome occurs in the setting of an active infection. This diversity was denoted by α and was measured using a diversity index using RNA-sequencing which showed more diversity for LRTI than non-LRTI enrolled patients.

Metagenomics next generation sequencing was next applied to integrate these three core elements. mNGS was used to identify microbial species. However, the presence of bacterial components in a blood specimen does not necessarily explain the cause of the patient's disease due to possibility of contamination or translocation of commensal bacteria to the bloodstream. In a similar manner, viral sequencing can detect clinically irrelevant or latent viruses in the bloodstream and thus would not explain the patient's disease. Therefore, complementing mNGS that detect microbes with host RNA transcript-based profiling using RNA signatures can provide better results in detecting infected patients, differentiating bacterial vs. viral infections.

The results of this integration resulted in a 36% reduction in antibiotics use, higher accuracy for identifying LRTI positive patients as compared to the standard of care. The detection of pathogens otherwise not usually tested for using classic viral PCR assays (i.e., influenzae C). The specificity and sensitivity of this assay were 87.5 and 100%, respectively. Therefore, the results of this study suggested using an integration protocol for these three elements of LRTI can operate as a promising and superior tool in the management and outcomes of LRTI patients (50–52).

Similarly, integrating mNGS for detecting bacterial DNA, host response profiling using previously defined host response transcript signatures and viral capture sequencing was performed in a prospective study of 200 patients enrolled from the ED with suspected sepsis. Study results show that each of the 3 techniques used showed an improvement of diagnosis of sepsis, and when used in combination, an even better improvement in diagnosis and management of sepsis was noted. One notable result of this study was that host response profiling led physicians to change their diagnostic decisions in 46 out of 100 patients highlighting the impact of host response profiling in the management of patients with suspected sepsis (52).

### Other Diagnostic Methods

Finally, the focus of this review is molecular assays based on the host immune response, although it is noteworthy to mention that advances in specific imaging modalities utilizing “omics” technology have contributed to improved microbial detection. A major step in the technological progress may be the implementation of 7T MRI imaging to investigate microbiological processes by sampling parameters of cell and tissue metabolism that are dynamic and subject to changes within certain cellular conditions such as infections (53).

## Discussion

A dynamic and temporal relationship between infectious processes and the host-immune response has been described in this review. Taking into consideration the impact of the host-response, attempts of using it as a reference for applying a more individualized approach of precision medicine has become the focus of many research studies. Using advanced assays that include RT-PCR, single cell RNA sequencing, mNGS, microarrays and RT-LAMP were reviewed and show high levels of accuracy compared to gold standard. Host-gene signatures, transcriptomics, proteomics, and expressed biomarkers used demonstrate promising results for a systematic integration of host immunodiagnostics with conventional microbial detection for improved management of infectious diseases. Host-based response may serve to be an alternative of the traditional time-consuming microbiological assays. However, a more holistic approach would be the integration of both host and pathogen-based diagnostics into one single platform. Future studies and clinical trials will be required to measure the true impact of combining these approaches.

One of the most important uses of the host-response as a tool for improving diagnostics has been the focus on the discrimination between bacterial and viral infections. Several studies were described in this review article that allow for the accurate discrimination of bacterial vs. viral etiologies in suspected infection. In fact, potential results of host-based diagnostics in this matter can achieve the WHO goal of ending tuberculosis in 2,035 if correctly implemented for superior pathogen diagnosis (27).

Accurate and rapid discrimination between bacterial and viral infections can also direct management by permitting proper antibiotics usage and prescription in a timely and directed manner. Ultimately, the improved patient outcome with higher and more rapid cure rates may translate into decreased mortality rate, healthcare costs for prolonged hospital stay, and the decrease in antibiotics misuse.

Host-based diagnostics have also shown major success in the diagnosis of sepsis. Considered a life-threatening process, sepsis calls for immediate life-saving intervention measures. Applying host-based diagnostics was shown to assist with the determination of the underlying etiology of sepsis as well as providing insight on the severity and prognosis.

Another important manifestation of host-immune diagnostics that has been highlighted in this review is the ability to distinguish latent infection from active infection as well as predicting the progression from latent to active infection at an earlier stage than standard microbiological tests. This should be an important aspect for future consideration that may necessitate a different approach with latent infections' management and prognosis.

Additionally, host gene signatures contribute to identification of treatment response over elapsed time as well as disease progression. This ability to measure response can prove to play an important role in determining staging of an infectious disease, its severity and its response to treatment.

This review also highlighted the potential of host-microbiota signatures to provide a perception of the severity and prognosis of certain infections. Although further future validation is required, such a link could facilitate the implementation of assays using microbial signatures to prognosticate respiratory infections concurrently with diagnostics for such infections.

From an economic perspective, host-based diagnostics may significantly reduce healthcare costs. Through improved definition of host response, more sensible use of antibiotics will ultimately lead to a reduction in the drug cost as well as antibiotic administration. Moreover, improved accuracy will likely lead to a decrease in the usage of consultation services as well as excessive procedures and laboratory tests being ordered (e.g., interventional radiology procedures, inflammatory markers, tumor markers, biopsies). These interventions may result in an overall decrease in cost on the individualized patient level and on the overall healthcare industry, although additional clinical trials will be required. In future applications, immunodiagnostics, unlike pathogen-based testing, may present the capability of differentiating non-infectious immune triggers including sterile inflammatory processes, autoimmune diseases, or malignancy. Further improvement in the currently existing platforms is required before such a claim can be translated into clinical practice and to possibly supersede and replace standard pathological techniques for such non-infectious causes.

One other advantage for host-response diagnostics as compared to pathogen-based diagnostics arises from the ability of viruses to mutate at a fast rate with emergence of different variants. Some RNA viruses can have a mutation rate up to a million times higher than their hosts and can incorporate mutated nucleotides at a rate of 10^−6^-10^−4^ substitutions per nucleotide per cell infection (54). This ability of viruses to rapidly mutate and transfer between hosts imposes a limitation for their detection and requires the development of dynamic means to detect current and emergent viral strains. Using the host as the diagnostic subject overcomes this limitation and proves yet another firm basis for adopting such methods.

**Figure 1:**
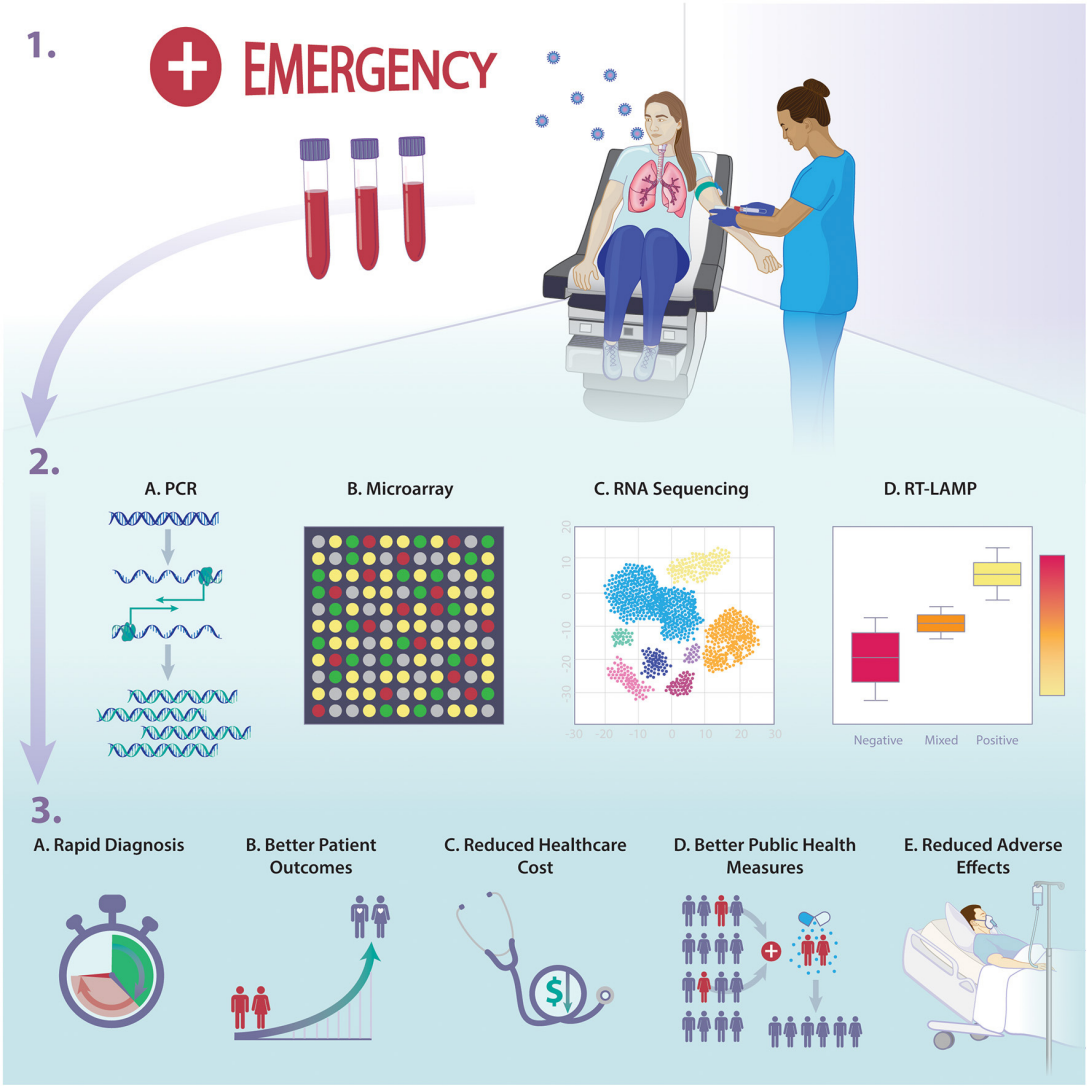
Overview of the implications of host-response diagnostics on infectious diseases management and outcome.

Finally, host-based diagnostics can help resolve multiple public health issues. A major effect of adopting more recent host-based diagnostics is preventing further AMR which is one of the most serious global public health threats. By providing accurate diagnostic outcomes, more precise and targeted therapies could be applied, thus, reducing the risk antibiotic overuse and the emergence of AMR. At a hospital, country or global scale, host-response diagnostics may also play a role in the rapid identification of exposure which can result in containment and improved infection control measures, especially in the setting of epidemics and/or pandemics. Several studies have shown more rapid results of testing the host response for infectious processes than traditional microbiology assays. This approach has proven to be essential in the case of viral infections with long incubation periods and those characterized with pre-symptomatic yet highly contagious phases. Using such assays could prove to help with infection containment during viral pandemics or influenza season. The early detection of the nature of an infectious disease would help aid determining which patients require early quarantine, and this would ultimately be reflected as better patient care during possible pandemics such as COVID-19 (7). Detecting such affected individuals will help in the quarantine process and put a limit for the transmission of diseases. The COVID-19 pandemic has uncovered the severe lack of means and the desperate need of public health measures that deal with phenomena of such impact and scale (see Figure 1).

## Potential Downfalls

As with other assays, host-based diagnostics have shortcomings. An important limitation in multiple studies is lack of adequate sample size and concern for appropriate power resulting in a possible increase in the margin of error. Larger cohorts in prospective studies are required to improve the robustness of study performance estimates.

Another limitation is the absence of special populations including immunocompromised hosts such as solid organ, stem cell transplant recipients and those individuals with autoimmune disorders. These patients are at risk for expanded infections including invasive fungal pathogens, which are not represented in current studies and require future investigation.

Among the drawbacks of host response-based diagnostics is the lack of precise identification of the pathogen involved. This prevents directed and specific treatment of the causative agent (19). Moreover, despite promising outcomes in differentiating bacterial vs. viral infections and thus limiting the use of antibiotics in case of viral infections, the lack of precise identification of the causative pathogen and ultimately the lack of isolation of such pathogen in the case of bacterial infection prevents the assessment of its sensitivity to antibiotics. This would impose a limitation to reducing antimicrobial resistance.

Cost and technical limitations exist to these assays. For RNA-sequencing techniques, high cost are major barriers to adoption, specifically in areas with limited resources. The development of inexpensive platforms would improve the prospects of more rapid utilization in healthcare setting. Additionally, some platforms are tuned to specific set of biomarkers, which make generalizability for detection of other diseases potentially difficult. Finally, microarrays are currently far too time-consuming with a turnaround time of about 1–2 weeks to be applied in a clinical setting (55). Thus, further laboratory validation should be attained before any of these assays can be used in clinical settings.

## Conclusion

This review describes multiple aspects of host-based response diagnostics as an adjunct to pathogen-based diagnostics and not as a replacement. However, favorable outcomes show that there are advantages of using host-based diagnostics as compared to pathogen-based diagnostics. Over 30 trials have focused on the use of host-response diagnostics for improved diagnosis of acute infection. Rapid and accurate diagnosis and prognosis can result in reduced healthcare costs, fewer adverse effects, reduction in antibiotic misuse and lower rates of antimicrobial resistance, improvement in public health measures for rapidly spreading endemics and pandemics, and ultimately better management with positive patient outcomes are potentials of adopting host immunodiagnostics. However, there remains some pitfalls including accessibility, cost, laboratory practicality and further clinical validation. While host immunodiagnostics show excellent promise, further investigations are needed to define the possible implications of adopting these novel modalities for the advancement in the field of infectious diseases.

## Author Contributions

JA drafted the original manuscript, responsible for article curation, and investigation. JA and MM revised and edited the draft. MM supervised and validated the final product. All authors gave final approval of this version to be published and agreed to be guarantor of the work.

## Funding

This work was supported, in part, by grants from the National Institute of Allergy and Infectious Diseases (RO1 AI132638) to MM.

## Conflict of Interest

MM reports consultation fees from Safi Biosolutions, Clear Creek Bio, Vericel, NED biosystems, GenMark Diagnostics, and Day Zero Diagnostics; grant support from Thermo Fisher Scientific and Genentech; medical editing/writing fees from UpToDate, outside the submitted work. MM also reports patents 14/110,443 and 15/999,463 pending. The remaining author declares that the research was conducted in the absence of any commercial or financial relationships that could be construed as a potential conflict of interest.

## Publisher's Note

All claims expressed in this article are solely those of the authors and do not necessarily represent those of their affiliated organizations, or those of the publisher, the editors and the reviewers. Any product that may be evaluated in this article, or claim that may be made by its manufacturer, is not guaranteed or endorsed by the publisher.

## Abbreviations

AMR: Antimicrobial resistance
AUROC: Area under the receiver operating characteristic curve
CAP: Community acquired pneumonia
PSI: Pneumonia severity index
RSV: Respiratory syncytial virus
RT-PCR: Real time polymerase chain reaction
LRTI: Lower respiratory tract infection
CRP: C-Reactive protein
FAM89A: Family With Sequence Similarity 89 Member A
IFI44L: Interferon Induced Protein 44 Like
mNGS: Metagenomics next generation sequencing
Tb: Tuberculosis
ED: Emergency Department
WHO: World Health Organization
NPV: Negative predictive value
PPV: Positive predictive value
PCT: Procalcitonin
IMKX-BWN-1: Inflammatix-bacterial-viral-non-infected-version 1
NAAT: Nucleic acid amplification test
RT-LAMP: Reverse transcription loop-mediated isothermal amplification
NTS: Nose and throat swabs
IP-10: Interferon-Inducible Protein 10
BCA-1: B-Cell attracting chemokine
OASL: Oligoadenylate synthetases-like
NP: Nasopharyngeal samples.
